# Evidence based practice profiles: Differences among allied health professions

**DOI:** 10.1186/1472-6920-10-69

**Published:** 2010-10-12

**Authors:** Maureen P McEvoy, Marie T Williams, Timothy S Olds

**Affiliations:** 1 School of Health Sciences, University of South Australia, North Tce, Adelaide, 5000, Australia

## Abstract

**Background:**

Most previous studies of allied health professionals' evidence based practice (EBP) attitudes, knowledge and behaviours have been conducted with profession specific questionnaires of variable psychometric strength. This study compared the self-report EBP profiles of allied health professionals/trainees in an Australian university.

**Methods:**

The Evidence-Based Practice Profile (EBP^2^) questionnaire assessed five domains (Relevance, Terminology, Practice, Confidence, Sympathy) in 918 subjects from five professional disciplines. One and 2-way factorial analysis of variance (ANOVA) and t-tests analysed differences based on prior exposure to EBP, stage of training, professional discipline, age and gender.

**Results:**

There were significant differences between stages of training (p < 0.001) for all domains and between EBP exposure groups for all but one domain (Sympathy). Professional discipline groups differed for Relevance, Terminology, Practice (p < 0.001) and Confidence (p = 0.006). Males scored higher for Confidence (p = 0.002) and females for Sympathy (p = 0.04), older subjects (> 24 years) scored higher for all domains (p < 0.05). Age and exposure affected all domains (p < 0.02). Differences in stages of training largely explained age-related differences in Confidence and Practice (p ≤ 0.001) and exposure-related differences in Confidence, Practice and Sympathy (p ≤ 0.023).

**Conclusions:**

Across five allied health professions, self-report EBP characteristics varied with EBP exposure, across stages of training, with profession and with age.

## Background

The provision of training in the five steps of evidence based practice (EBP): ask, acquire, appraise, apply and analyse/adjust, are seen as the key skills required for life-long learning for professional decision-making [1]. While much of the early drive toward incorporating evidence into clinical practice occurred in medicine (evidence- based medicine approach), this concept now extends to allied health and social care professions [1]. While the past focus for EBP has been training of health graduates within the health workplace, more recently the importance of embedding training and evaluation of skills in EBP into undergraduate curricula, has been recognised [2].

With longer life expectancy, availability of advanced medical care and associated rising health care costs, EBP has been embraced by governments around the world. This provides 'checks and balances' between researchers and clinicians to inform each other in the provision of efficient and effective health care with a flow-on effect into medical and health training institutions. Implementing EBP in curricula is not a matter of choice for universities: it has become a necessity. There are good pedagogical reasons for embedding EBP within curricula with much emphasis on the teaching-research nexus and the need to provide opportunities for research to inform and underpin both the *content *of what is taught, and the *way *in which it is taught.

Appropriate training in knowledge, attitudes and skills are the foundation for an EBP approach. While training and assessment of knowledge and skill components of EBP can be achieved in a number of ways, the attitudinal/perceptual component to EBP is less clearly defined. Dawes et al. (2005) have suggested that attitudes towards EBP are 'caught' rather than 'taught', as part of immersion in the professional culture and cannot be entirely directed or dictated by curricula.

There are an abundance of studies concerning allied health professionals' self-reported attitudes, knowledge and behaviours toward EBP. The majority of studies have been conducted within individual professions, such as physiotherapy, occupational therapy, medical radiation and podiatry [3-13]. Studies across health professional disciplines are less common [14-19] and subgroup analysis among professions is rare [16-19]. Rather than the clinical use of EBP, Metcalfe et al. (2001) and Pain et al. (2004) compared discipline groups (speech therapists, occupational therapists and physiotherapists) for the perceived importance of research and for orientation toward research respectively. To date, there appears to be only one study which specifically compared knowledge and use of EBP among practising allied health professionals [19]. Upton and Upton (2006) reported significant differences in knowledge and use of EBP among professions but prior exposure and training in EBP was not explored as an explanatory variable.

Collating information about an individual's behaviours, beliefs and attitudes has been used to construct profiles of individuals in the finance and law enforcement arenas (credit or criminal profiles) [20]. When sufficient numbers of profiles are available, analysis of patterns within the data may predict future behaviours. An individual's 'EBP profile' may be constructed via responses in domains commonly associated with EBP: knowledge, confidence, behaviours and attitudes. The aim of the current study was to explore the influences of a range of characteristics on an individual's EBP profile. While there are many characteristics that may influence an EBP profile, this study prospectively explored the influence on EBP profiles of the primary variables of prior exposure to EBP training, stage of professional training (undergraduate years, post-graduate) and professional allied health disciplines (physiotherapy, occupational therapy, podiatry, medical radiation, human movement) with age and gender explored as secondary variables.

In this study, the primary research question was: Do prior exposure to EBP, stage of training, and professional health discipline, influence the EBP profile? Secondary analysis considered the influence of age and gender.

## Methods

### Overview

This cross-sectional, descriptive study recruited academic staff and students at a large Australian university across a number of health sciences disciplines and stages of training, to complete a self-report questionnaire. To enable maximum recruitment the questionnaire was delivered face-to-face (paper-based) to students and was also available in both paper and online versions for completion by staff. Ethical approval for this study was granted by the Human Research Ethics Committee of the University of South Australia.

### Sample

Recruitment procedures were designed to provide an opportunity for all academic staff and students of five allied health professional disciplines to complete the questionnaire. At the time of the study, there were 120 full and part-time academic staff and 1676 full and part-time undergraduate students in the School of Health Sciences, University of South Australia dispersed across bachelor degree programs in physiotherapy, occupational therapy, podiatry, medical radiation and human movement. These five degree programs included varying exposures to research training (statistics and research methods) and EBP.

### Study Questionnaire

The Evidence-Based Practice Profile (EBP^2^) takes 10-12 minutes to complete and consists of a total of 58 items, each using a 5-point Likert scale, with a further 13 items which address demographic characteristics including age, gender, professional discipline, stage of training (year level for undergraduates or post-graduate) and prior exposure to EBP training. The questionnaire includes five domains (Relevance, Terminology, Confidence, Practice, Sympathy) (McEvoy et al. 2010). Relevance (14 items) refers to the value, emphasis and importance placed on EBP; Terminology (17 items) refers to the understanding of common research terms; Confidence (11 items) refers to the perception of an individual's abilities with EBP skills; Practice (9 items) refers to the use of EBP in clinical situations and Sympathy (7 items) refers to the individual's perception of the compatibility of EBP with professional work. Psychometric testing during the development of the EBP^2 ^confirmed test retest reliability (intraclass correlation coefficients ranging from 0.77 to 0.94), internal consistency (Cronbach's alpha 0.96) and validity (convergent validity Pearson correlation coefficient for domains ranging from r = 0.54-0.80) [21]. Responses to the EBP^2 ^were able to distinguish groups with different levels of prior EBP exposure for three of the five domains of the instrument (Relevance, Terminology and Confidence, ANOVA *p *< 0.001 to 0.004).

### Procedure

Program directors in five health disciplines (physiotherapy, podiatry, occupational therapy, medical radiation and human movement) were contacted and asked to identify a class in each year level where students could be invited to complete a paper version of the questionnaire. All students were sent an information sheet prior to this class. All academic staff were invited to complete a paper version of the questionnaire at a routine monthly meeting where an inventory of attendance was made. Academics not in attendance were followed up by email and provided with an opportunity to complete the questionnaire by paper or an electronic version. All returned questionnaires remained anonymous with identification codes used for distinguishing staff and programs.

### Data management

All data management and analysis was undertaken using SPSS Statistics 17.0 (Chicago, IL). Data were entered or imported into SPSS and checked for missing values. While there are a number of mathematical approaches to address missing data, hot deck imputation has been reported amongst those recommended [22]. Missing values were imputed using the "Hot Deck" method where there was completion of at least 75% of the non-demographic items. This involved filling missing data responses with responses determined from the most similar complete records. Where more than 25 percent of the non-demographic items were not complete the record was excluded from further analysis.

Participants were categorised to form subgroups based on exposure to EBP (no exposure, ≤ 20 h, > 20 h), stage of training (1^st^, 2^nd^, 3^rd^/4^th ^combined undergraduate, post-graduate), disciplines (physiotherapy, podiatry, occupational therapy, medical radiation, human movement), age ≤ 24 yrs, > 24 yrs) and gender (female, male). Due to the small number of undergraduate students completing a 4^th ^year of their degree (only in physiotherapy) the 3^rd ^and 4^th ^year groups were combined. For the subgroup analysis of stage of training, graduates and staff subjects were referred to as 'post-graduate'. The age categories aimed to separate subjects currently enrolled or who had previously completed bachelor degrees, with 24 years chosen, as this is the age at which most Australians continuing university studies direct from school (± a chosen deferred year), will have completed an entry level degree.

One-way factorial analysis of variance (ANOVA), t-tests and post hoc tests [Tukey's Honestly Significant Difference (HSD) test] were used to analyse differences among the levels of the primary variables (prior EBP exposure, stage of training, professional discipline) and secondary variables (age and gender) on the EBP domain scores for Relevance, Terminology, Confidence, Practice and Sympathy. Significance was set at p < 0.05. Because there were likely to be strong correlations among age, level of exposure and stage of training, two-way factorial ANOVAs were used where appropriate to explore possible confounding variables. Because of missing cells, it was not possible to perform a three-way ANOVA.

## Results

### Response Rate

The EBP^2 ^was completed by 933 subjects of which 898 were students from a potential 1676 students enrolled in the included bachelor programs (student response rate 54%). The specific bachelor program was known for 884 of these students. Based on enrolled bachelor students in the discipline programs, this constituted 60% in physiotherapy, 50% in podiatry, 63% in occupational therapy, 71% in medical radiation and 39% in human movement. The questionnaire was completed by 35 of 120 post-graduates (mostly academic staff giving a response rate 29%). There was > 25% of incomplete data for 15 subjects (14 students and one post-graduate) and these records were excluded from any further analysis. In the final dataset of 918 included for analysis, there were 884 students and 34 post-graduates.

### Respondent characteristics

The demographics (age, gender, prior EBP training exposure, stage of training and discipline groups) of the sample are presented in Table 1.

**Table 1:** Demographic details (age, gender, EBP training, stage of training and discipline groups) of the sample

	n =	%
**Age**		
Number completing item	905	99
Mean Age (SD) range	22 (7) 17-59 years	
		
**Gender**		
Number completing item	887	97
Females	634	69
Males	253	28
		
**EBP training**		
Number completing item	880	96
No training	618	67
Single lecture (1-3 h)	84	9
Weekend course (3-10 h)	6	1
Short course (10-20 h0	16	2
University course (> 20 h)	156	17
		
**Stage of training**		
Number completing item	915	99.7
1^st ^year	236	26
2^nd ^year	300	33
3^rd ^year/4^th ^year	345	38
Post-graduate	34	3
		
**Discipline groups**		
Number completing item	887	97
Physiotherapy	242	26
Podiatry	44	5
Occupational Therapy	171	19
Medical Radiation	173	19
Human Movement	257	28

**Figure 1:**
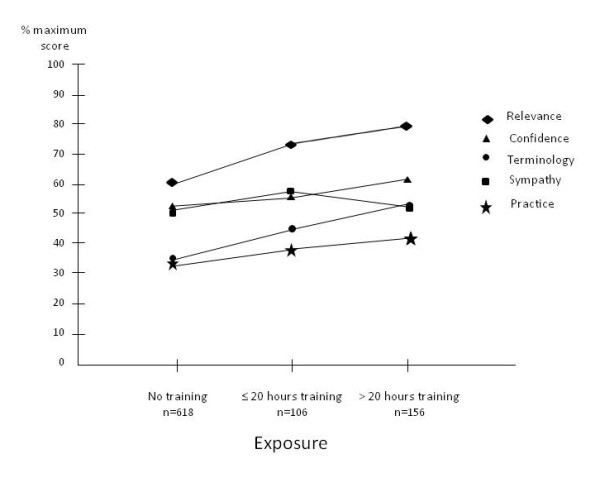
**The percentage score (of maximum) for each domain for the levels of exposure**.

The mean (SD) scores for each domain of the EBP^2 ^and significant findings for exposure, stage of training and professional discipline groups are presented in Table 2. There was a positive relationship between increasing exposure and scores for the EBP domains of Relevance and Terminology. Scores for Confidence were significantly different for respondents with no exposure and greater than 20 hours exposure. Scores for Practice improved significantly after less than 20 hours exposure but there was no significant difference between less than and greater than 20 hours exposure. The percentage score (of maximum) for each domain for the levels of exposure are presented in Figure 1.

**Table 2:** Mean (SD) and p values for factorial ANOVA for three EBP training groups (n = 880), for four stages of training (n = 915) and for five discipline groups (n = 887) for Relevance, Terminology, Confidence, Practice and Sympathy.

	Domain (max possible score)				
	Relevance (70) mean (SD)	Terminology (85) mean (SD)	Confidence (55) mean (SD)	Practice (45) mean (SD)	Sympathy (35) mean (SD)
**Prior exposure to EBP**					
No training n = 618	49 (10)^a^	40 (12)^a^	34 (8)^a^	21 (7)^ab^	21 (3)^a^
≤ 20 h training n = 106	55 (8)^a^	47 (13)^a^	35 (6)	23 (5)^a^	22 (4)^ab^
> 20 h training n = 156	58 (8)^a^	52 (11)^a^	37 (8)^a^	24 (7)^b^	21 (4)^b^
p value	< 0.001	< 0.001	< 0.001	< 0.001	0.003
**Stage of training**					
1^st ^year n = 236	45 (8)^a^	36 (13)^a^	34 (7)^a^	20 (7)^a^	21 (3)^a^
2^nd ^year n = 300	52 (10)^a^	43 (11)^a^	34 (8)^b^	22 (7)^a^	21 (4)^b^
3^rd^/4^th ^year n = 345	54 (9)^a^	46 (12)^a^	35 (8)^c^	21 (6)^b^	21 (4)^c^
Post-graduate n = 34	61 (7)^a^	60 (15)^a^	40 (10)^abc^	27 (7)^ab^	26 (4)^ab c^
p value	< 0.001	< 0.001	< 0.001	< 0.001	< 0.001
**Discipline groups**					
Physiotherapy n = 242	56 (8)^abc^	50 (11)^abc^	35 (8)	22 (6)^abc^	21 (4)
Podiatry n = 44	53 (10)^de^	47 (14)^def^	37 (7)^a^	22 (6)^d^	21 (4)
Occupational Therapy n = 171	53 (9)^afg^	40 (12)^ad^	35 (8)	25 (7)^adef^	21 (3)
Medical Radiation n = 173	48 (8)^bdf^	40 (11.0)^be^	35 (7)	19 (6)^be^	21 (3)
Human Movement n = 257	46 (10)^ceg^	38 (13)^cf^	33 (8)^a^	20 (6)^cf^	21 (3)
p value	< 0.001	< 0.001	0.006	< 0.001	0.802

Groups with the same superscript are significantly different to each other within the same domain. For example, in stage of training groups for Confidence, post-graduates scored significantly higher than 1^st^, 2^nd ^and 3^rd^/4^th ^years but the latter three groups did not score significantly differently to each other.

**Figure 2:**
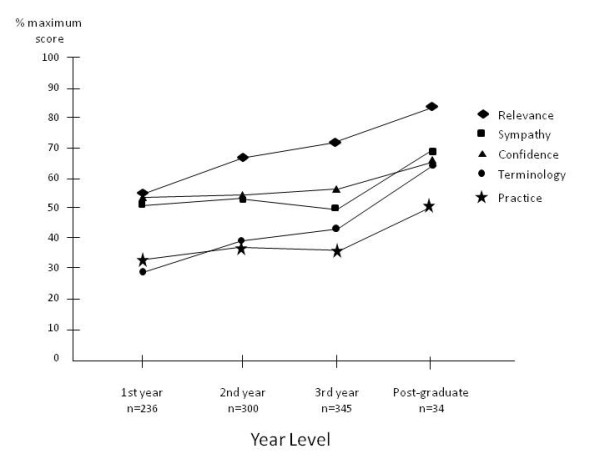
**The percentage score (of maximum) for each domain for the stages of training**.

Scores for the EBP domains of Relevance and Terminology increased significantly with each year of progression of undergraduate training, with scores for the domain of Practice differing significantly between the first and second years of programs and between the 3^rd^/4^th ^and post-graduate levels but not between the 2^nd ^and 3^rd^/4^th ^year levels. There was no significant difference between scores for the domains of Confidence and Sympathy among undergraduate stages of training, though scores were significantly greater for subjects who had completed training (post-graduate). The percentage scores for each domain for the stages of training are presented in Figure 2.

**Figure 3:**
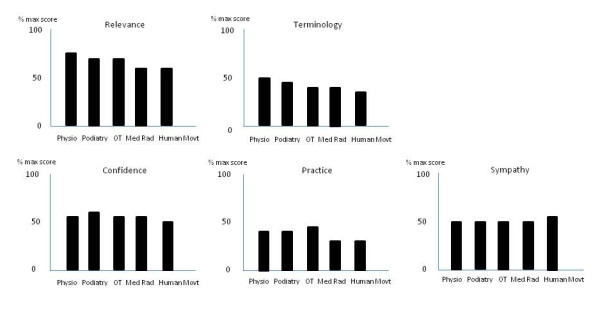
**The percentage score (of maximum) for each domain for the professional discipline groups (physiotherapy n = 242, podiatry n = 44, occupational therapy n = 171, medical radiation n = 173, human movement n = 257)**.

There were a number of differences among professional disciplines when all subjects (students and post-graduates) were combined. Physiotherapists had significantly higher scores for Relevance compared to all other disciplines except podiatrists. Podiatrists and occupational therapists scored significantly higher for Relevance compared to medical radiation and human movement subjects. For Terminology, physiotherapist and podiatrists scored significantly higher than occupational therapists, medical radiation and human movement subjects. For Confidence all disciplines scored similarly except that podiatrists had a significantly higher score than human movement subjects. For the domain of Practice, occupational therapists scored significantly higher than all other disciplines, with physiotherapists scoring significantly higher than medical radiation and human movement subjects. There was no significant difference across the disciplines for the domain of Sympathy. The percentage score (of maximum) for each domain for the professional discipline groups are presented in Figure 3.

Mean and SD values for domain scores for age and gender are presented in Table 3. For each EBP domain, scores for the older respondents (> 24 years) were significantly higher than respondents ≤ 24 years. In the gender groups, scores for the EBP domain of Confidence and Sympathy were significantly different between males and females with males scoring Confidence higher than females and females scoring Sympathy higher than males. There was no significant difference between females and males for the three remaining EBP domains of Relevance, Terminology and Practice.

**Table 3:** Mean (SD) and p values for independent t-tests for two age groups (n = 724), for gender (n = 898) for the 5 domains (Relevance, Terminology, Confidence, Practice, Sympathy)

Domain (max possible score)	Relevance (70) mean (SD)	Terminology (85) mean (SD)	Confidence (55) mean (SD)	Practice (45) mean (SD)	Sympathy (35) mean (SD)
**Age groups**					
17-24 n = 736	50 (10)	42 (12)	35 (8)	21 (6)	21 (6)
> 24 YRS n = 149	57 (9)	49 (15)	36 (9)	23 (7)	23 (4)
p value	< 0.001	< 0.001	0.045	0.002	< 0.001
**Gender**					
Female n = 645	52 (10)	43 (13)	34 (8)	22 (7)	22 (4)
Male n = 253	50 (10)	44 (14)	36 (8)	21 (7)	21 (4)
p value	0.05	0.09	0.002	0.23	0.04
p value	0.48	0.32	0.64	0.96	0.37

While significant differences existed between domain scores for age, exposure and stage of training categories, these differences were potentially confounded by the strong inter-correlations between these three variables. To test this hypothesis, three 2-way ANOVAs were conducted with age and exposure levels, age and stage of training and stage of training and exposure levels as grouping domains.

Significant main effects were evident for age and exposure for all five domains (Relevance: p < 0.001 and p < 0.001 respectively; Terminology: p < 0.001 and p < 0.001; Confidence: p = 0.019 and p < 0.001; Practice: p = 0.005 and p < 0.001; Sympathy: p < 0.001 and p < 0.001).

Significant main effects were evident for age and stage of training for the domains of Relevance (p < 0.001 and p < 0.001 respectively), Terminology (p = 0.025 and p < 0.001), and Sympathy (p = 0.012 and p < 0.001). For Confidence and Practice, there was a significant main effect for stage of training only (p < 0.001), and the main effect for age did not reach statistical significance.

Significant main effects were evident for stage of training and EBP exposure for Relevance (p < 0.001 and p < 0.001 respectively) and Terminology (p < 0.001 and p < 0.001). For Confidence, Practice and Sympathy there was a significant main effect for stage of training (p = 0.023, p < 0.001 and p < 0.001 respectively) but the main effect for EBP exposure did not reach statistical significance.

## Discussion

### Strengths and limitations

This is the first study to demonstrate that the self-report EBP profile differs with the degree of prior exposure to formal EBP training and with the stages of training (undergraduate and post-graduate) and differs across allied health professional disciplines. While the best teaching practices and processes for EBP remain unclear, the findings in this study support early and repeated exposure to provide the optimal opportunities for reinforcing an EBP approach. The study was not designed to explore why the differences exist (across stages of training and professions and exposures) or to suggest how EBP should be taught but the findings provide a cross-sectional description of predominantly undergraduate allied health EBP profiles in one Australian university. As they are likely to reflect the culture of this university, at this stage the findings should not be generalised to the EBP culture of health professionals at a national or international level. While providing an insight into the EBP profile of a large school within one university at one point in time there is obviously value in conducting future longitudinal studies, comparative studies and studies incorporating larger non-student populations.

### Primary variables, pattern and comparison with previous studies

It is possible that the changes in domain scores could be affected by the time that elapses while taking EBP courses, time while progressing through the years of a university program or just getting older. Notwithstanding the possibility of these secular contributions to the cross-sectional findings, the consistent gradients between age, exposure and stage of training, and the domains of Relevance and Terminology, suggest that EBP training can positively affect these domains. Confidence in the skills of EBP demonstrated fewer differences across stages of training. Upton and Upton (2006) reported similar findings and suggested that the actual skills may not match the perception of abilities. In the current study one can only speculate that confidence levels may mask a lack of awareness of limitations in skills in the early years of training on the one hand, and lack of acknowledgement of advancement in skills in the later years of a program on the other hand, that is, 'the more you know, the more you realise you don't know'.

Different professional 'strengths' were reflected in the current study in higher domain scores for attitudes, knowledge and practice of EBP across professions (occupational therapists reported higher scores for Practice, physiotherapists for Relevance, physiotherapists and podiatrists for Terminology). While the Canadian study by Pain et al (2004) focussed on research rather than EBP, speech therapists placed significantly greater value on research and its use in clinical practice than physiotherapists and occupational therapists. In addition, while physiotherapists were least likely to have undertaken formal research training and had the greatest number of years in practice, this did not translate to any difference in time spent on involvement in research activities (eg grant writing, data collection and analysis, and research presentation) when compared to the other professions [17]. In a United Kingdom study, Upton and Upton (2006), reported significant differences in knowledge and use of EBP between 14 professional disciplines including physiotherapists (n = 98), occupational therapists (n = 86), podiatrists (n = 20) and radiographers (n = 70). Confidence in research skills, and application of these in general, rated more poorly in these four professions when compared to professions such as medical physicists and psychologists. There were no data reported by Upton and Upton (2006) on prior exposure to EBP training.

### Secondary variables, pattern and comparison with previous studies

Not unexpectedly, older subjects scored higher than younger subjects for each domain of the EBP^2 ^questionnaire. It is likely that with increasing years, greater potential for both formal and informal exposure to EBP training may have occurred. The findings from the two-way factorial ANOVAs suggested that while age and exposure both affected all factors, age-related differences in Confidence and Practice were largely explained by differences in stages of training, and exposure-related differences in Confidence, Practice and Sympathy were largely explained by differences in stage of training. Stage of training, perhaps because it combines maturity and exposure, may be the key variable. To date, gender differences with respect to EBP have not been reported. Our findings indicated that males were significantly more confident in their EBP knowledge and skills while females were more positive in their attitudes and sympathy to EBP.

### Interpretation

While a broad measure of prior exposure to EBP training was included in the EBP^2 ^questionnaire, the main limitations to interpreting the findings stem from the lack of detail concerning the nature of this prior EBP exposure. There were significant differences in the EBP profile for participants who had > 20 hours formal training, differences among stages of training for undergraduate students and differences among disciplines. Within the curriculum for each of the disciplines included in this study, EBP training was included in a variety of modes but there was no consistent curriculum or pedagogical approach. For example, at the time of this study, the physiotherapy degree included a core EBP course within the curriculum while all other professional disciplines disseminated EBP training and philosophy across a number of courses. However, each professional discipline demonstrated high scores in at least one of the EBP domains suggesting that knowledge or attitudinal changes resulted from the EBP training curriculum.

It is tempting to suggest that the higher scores in EBP Relevance and in knowledge of EBP Terminology reflect more effective training for these characteristics in current curricula than the characteristics of Confidence and Practice of EBP. It may also be that Confidence and Practice are not able to be effectively taught within the formal university curriculum and change in these domains is more likely to be influenced by consistent immersion in clinical practice. The impact of professional socialisation on undergraduate students' attitudes and experience is difficult to gauge. In concert with exposure to academics within their respective professions, allied health students undertake clinical practice/field work in a range of settings (hospitals, schools, community centres, health centres) and will come into contact with a large variety of people (clinicians, supervisors, administrators, patients). In these encounters, the attitudes and behaviours of individual staff toward evidence-based practice is likely to be highly variable in terms of empathy, approval and understanding of EBP. This again takes us back to the question: "Is EBP taught or caught?" For Confidence, Practice and Sympathy it appears that both may contribute.

### What needs to be done now?

These observed cross-sectional relationships need to be complemented by longitudinal studies to allow for separation of genuine self-report changes from those which may be secular patterns. There is a need also to explore the relationship between the nature of EBP training in the undergraduate curriculum (in relation to the total time spent in formal training, the content, the timing of this training through a program, the format, assessment etc) and the characteristics of a self-report profile (Relevance, Knowledge, Confidence, Practice and Sympathy). Once this is known, intervention studies into the effects of training on EBP profiles can be conducted, and it may be possible to identify areas responsive and unresponsive to formal training.

## Conclusions

Growing evidence suggests that EBP is making in-roads into allied health. There is currently little existing literature however on the differences among health professions in terms of their knowledge, attitudes or practice of EBP. This study shows clear cross-sectional differences. Self-report knowledge, attitudes and behaviours vary with exposure, chosen profession, progression through a program and with age but there is no clear indication about which domains may be modified to bring about effective life-long learning in EBP. If the ultimate aim of EBP training is to have all allied health professionals confident, knowledgeable and skilled in the practice of EBP, greater effort needs to be exerted in determining how best to deliver the training, how much training, content, length and positioning of training courses throughout programs of study.

## Competing interests

The authors declare that they have no competing interests.

## Authors' contributions

MPM, MTW, TSO all instigated and conceived of the study, participated in the design of the study, participated in development and testing of the questionnaire, co-ordinated data collection and edited the manuscript. All authors read and approved the final version.

## Authors' information

MPM is a Lecturer, School of Health Sciences, University of South Australia and PhD candidate. Her research interests are in education of undergraduate health sciences students, teaching and curricula development and implementation of EBP.

MTW is an Associate Professor, School of Health Sciences, University of South Australia. Her research interests include evidence based practice education with particular reference to educational processes for best research evidence and sensation of breathlessness in chronic pulmonary conditions. 

TSO is a Professor of Health Sciences at the University of South Australia. His research interests include the links between time use and health outcomes, mathematical modelling of sports performance, anthropometry and historical trends in fitness, fatness, food intake and sleep of children

## Pre-publication history

The pre-publication history for this paper can be accessed here:

http://www.biomedcentral.com/1472-6920/10/69/prepub
